# Intermittent post-exercise sauna bathing improves markers of exercise capacity in hot and temperate conditions in trained middle-distance runners

**DOI:** 10.1007/s00421-020-04541-z

**Published:** 2020-11-19

**Authors:** Nathalie V. Kirby, Samuel J. E. Lucas, Oliver J. Armstrong, Samuel R. Weaver, Rebekah A. I. Lucas

**Affiliations:** 1 University of Birmingham, Birmingham, B15 2TT UK; 2 Performance Centre, University of Birmingham Sport, Birmingham, UK

**Keywords:** Post-exercise sauna, Thermoregulation, Exercise performance, Heat acclimation, Ergogenic aid

## Abstract

**Purpose:**

This study investigated whether intermittent post-exercise sauna bathing across three-weeks endurance training improves exercise heat tolerance and exercise performance markers in temperate conditions, compared to endurance training alone. The subsidiary aim was to determine whether exercise-heat tolerance would further improve following 7-Weeks post-exercise sauna bathing.

**Methods:**

Twenty middle-distance runners (13 female; mean ± SD, age 20 ± 2 years, $V$O_2max_ 56.1 ± 8.7 ml kg^−1^ min^−1^) performed a running heat tolerance test (30-min, 9 km h^−1^/2% gradient, 40 °C/40%RH; HTT) and temperate (18 °C) exercise tests (maximal aerobic capacity [$V$O_2max_], speed at 4 mmol L^−1^ blood lactate concentration ([La^−^]) before (Pre) and following three-weeks (3-Weeks) normal training (CON; *n* = 8) or normal training with 28 ± 2 min post-exercise sauna bathing (101–108 °C, 5–10%RH) 3 ± 1 times per week (SAUNA; *n* = 12). Changes from Pre to 3-Weeks were compared between-groups using an analysis of co-variance. Six SAUNA participants continued the intervention for 7 weeks, completing an additional HTT (7-Weeks; data compared using a one-way repeated-measures analysis of variance).

**Results:**

During the HTT, SAUNA reduced peak rectal temperature (*T*_rec_; − 0.2 °C), skin temperature (− 0.8 °C), and heart rate (− 11 beats min^−1^) more than CON at 3-Weeks compared to Pre (all *p* < 0.05). SAUNA also improved $V$O_2max_ (+ 0.27 L^−1^ min^−1^; *p* = 0.02) and speed at 4 mmol L^−1^ [La^−^] (+ 0.6 km h^−1^; *p* = 0.01) more than CON at 3-Weeks compared to Pre. Only peak *T*_rec_ (− 0.1 °C; *p* = 0.03 decreased further from 3-Weeks to 7-Weeks in SAUNA (other physiological variables *p* > 0.05).

**Conclusions:**

Three-weeks post-exercise sauna bathing is an effective and pragmatic method of heat acclimation, and an effective ergogenic aid. Extending the intervention to seven weeks only marginally improved *T*_rec_.

**Electronic supplementary material:**

The online version of this article (10.1007/s00421-020-04541-z) contains supplementary material, which is available to authorized users.

## Introduction

Heat acclimation improves exercise performance in the heat (Sawka et al. 2011). The most common model studied in the literature is “medium-term” active heat acclimation (Tyler et al. 2016), where individuals exercise in a climatic/environmental chamber for 60–120 min for 7–14 consecutive days (Garrett et al. 2009). This model of heat acclimation carries considerable barriers related to financial and temporal costs, as well as accessibility to climatic/environmental chambers. Consequently, heat acclimation is not widely used by athletes (recently recorded prevalence of ~ 15%; Périard et al. 2015) despite its effectiveness in improving exercise performance in hot environments. With major athletic events in extreme heat fast approaching, such as the Tokyo 2020 Olympic Games (postponed; Gerrett et al. 2019) and the 2022 FIFA World Cup in Doha, it is imperative that athletes are prepared to compete in such challenging environments. Post-exercise sauna bathing presents a practical and accessible heat acclimation alternative to active heat acclimation that could be implemented without disruption to an athlete’s training programme. Repeated bouts of sauna bathing have been observed to elicit some heat acclimation adaptations [e.g., reduced resting core temperature with sauna independent of exercise (Leppäluoto et al. 1986) and plasma volume expansion using post-exercise sauna bathing (Scoon et al. 2007; Stanley et al. 2015)]. However, its efficacy to elicit hallmark heat acclimation adaptations during exercise heat stress (e.g., reduced exercising heart rate and body temperatures, etc.; Sawka et al. 2011) has not previously been investigated (Casadio et al. 2017). Despite the relatively sparse scientific evidence, post-exercise sauna bathing is recommended to athletes preparing for competition in the heat (Racinais et al. 2019). It more specifically recommends that ahtletes use post-exercise sauna bathing to prepare for competition in the heat than the reference currently used. Therefore, the first aim of this study was to assess exercise heat tolerance following repeated bouts of intermittent sauna bathing using a fixed-workload exercise heat stress test.

The myriad of physiological adaptations attained through heat acclimation may improve exercise performance in cool or temperate conditions (Minson and Cotter 2016). Similar improvements may result from post-exercise sauna bathing, as a case study of a young female tennis player showed improvements in performance outcomes of time-to-exhaustion (TTE), maximal aerobic capacity ($V$O_2max_), and lactate threshold after 12 sauna sessions following exercise spread across 3 weeks (Novak et al. 2018). Similarly, Scoon and colleagues (2007) observed that running TTE increased in six male athletes after approximately 12 intermittent post-exercise sauna sessions. However, the effects of intermittent post-exercise sauna bathing on more objective markers of temperate exercise performance (i.e., $V$O_2max_ and blood lactate profile) have not been assessed in a larger cohort. Therefore, the second aim of this study was to assess whether integrating intermittent post-exercise sauna bathing (~ 3 sessions·week^−1^) into an endurance training programme further improves temperate exercise performance markers ($V$O_2max_ and running speed at 4 mmol L^−1^ blood lactate concentration [La^−^]), as compared to an endurance training programme alone.

Finally, long-term heat acclimation interventions (i.e., > 14 days) are expected to maximise exercise capacity in the heat (Tyler et al. 2016). These longer protocols may also allow time for the increased production of erythropoietin (EPO) and vascular endothelial growth factor (VEGF), as observed following hypoxia exposure (Girard et al. 2013), both primary factors driving favourable adaptations to oxygen carrying capacity and delivery. Notably, 10 consecutive days of active heat acclimation elicits increases in hypoxia inducible factor 1-alpha (HIF-1a; the master regulator of downstream oxygen-modulating genes such as EPO and VEGF) in males (Lee et al. 2016). Therefore, the subsidiary aim of this study was to assess exercise heat tolerance and changes in EPO and VEGF in response to long-term (i.e., more than 14 heat exposures) intermittent post-exercise sauna bathing.

The purpose of this study was to determine the efficacy of adding intermittent post-exercise sauna bathing to normal endurance training to induce heat acclimation adaptations in trained athletes, and to determine the impact of these adaptations on maximal and submaximal exercise performance markers in temperate conditions. We hypothesised that intermittent post-exercise sauna bathing would: (1) elicit hallmark heat acclimation adaptations and improve thermoregulation during exercise heat stress more than endurance training alone, and (2) improve physiological markers of exercise performance in temperate conditions to a greater extent than endurance training alone.

## Methods

### Ethical approval

This study was approved by the University of Birmingham Ethics Committee (ERN_18-0958), and conformed to the standards set by the Declaration of Helsinki. All participants were informed of the experimental procedures and possible risks involved in the study before providing written consent.

### General overview and design

**Fig. 1:**
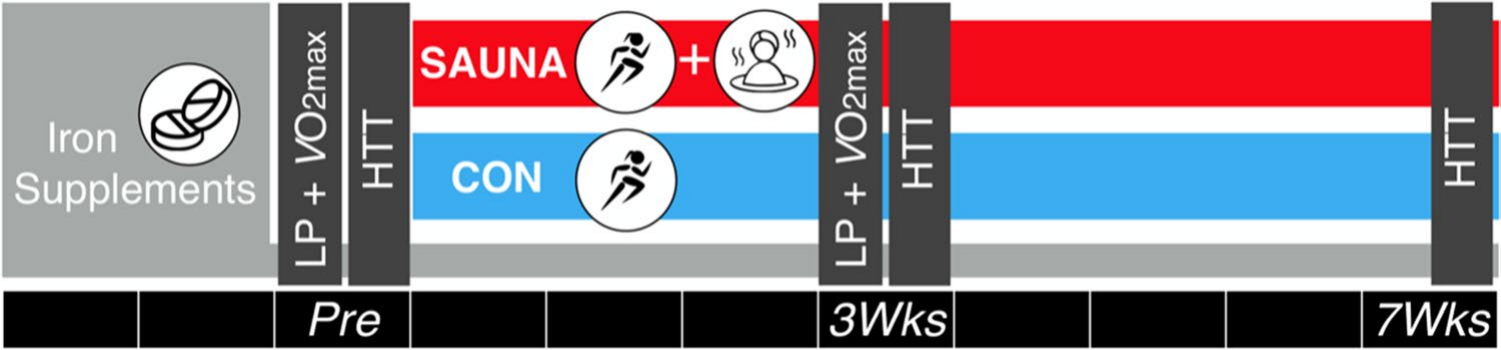
Schematic of the experimental design. Post-exercise sauna bathing intervention group (SAUNA) and control group (CON) completed temperate exercise tests (18 °C) consisting of lactate profile (LP) and maximal aerobic capacity ($V$O_2max_) tests, and a running heat tolerance test (HTT; 30-min, 9 km h^−1^/2% gradient, 40 °C/40%RH), at baseline (Pre), and following 3-Weeks (3Wks) intervention or control. Participants completed an additional HTT following 7-Weeks intervention (7Wks). Lower black bars indicate weeks

Each participant completed a general health questionnaire and female participants completed a menstrual cycle questionnaire. Throughout the protocol, participants were asked to keep a training diary (including session type, running distance, frequency and session perceived exertion) and female participants were asked to record their menstrual cycle. An overview of the protocol is depicted in Fig. 1. Participants took daily iron tablets (65 mg ferrous sulfate; Nature Made, West Hills, CA, United States) from 2 weeks prior to the first experimental trial until the completion of the protocol.

A battery of experimental trials was completed within a 1-week timeframe at baseline (Pre), following 3 weeks (3-Weeks) and following 7 weeks (7-Weeks) of either normal endurance training (CON) or normal endurance training with the post-exercise sauna bathing intervention (SAUNA). Experimental trials included a running heat tolerance test (HTT), a temperate exercise test (consisting of a lactate profile test to determine running speed at 4 mmol L^−1^ [La^−^] and a $V$O_2max_ test) and a resting venous blood sample. The temperate exercise test was conducted at Pre and 3-Weeks only.

Experimental trials following three-and seven-weeks intervention were arranged ~ 28 days apart to test female participants during the same menstrual cycle phase, as per a typical menstrual cycle. Participants self-allocated into groups prior to Pre testing. This experiment was conducted in the United Kingdom between the months of October and March to minimise any natural heat acclimatisation.

### Participants

**Fig. 2:**
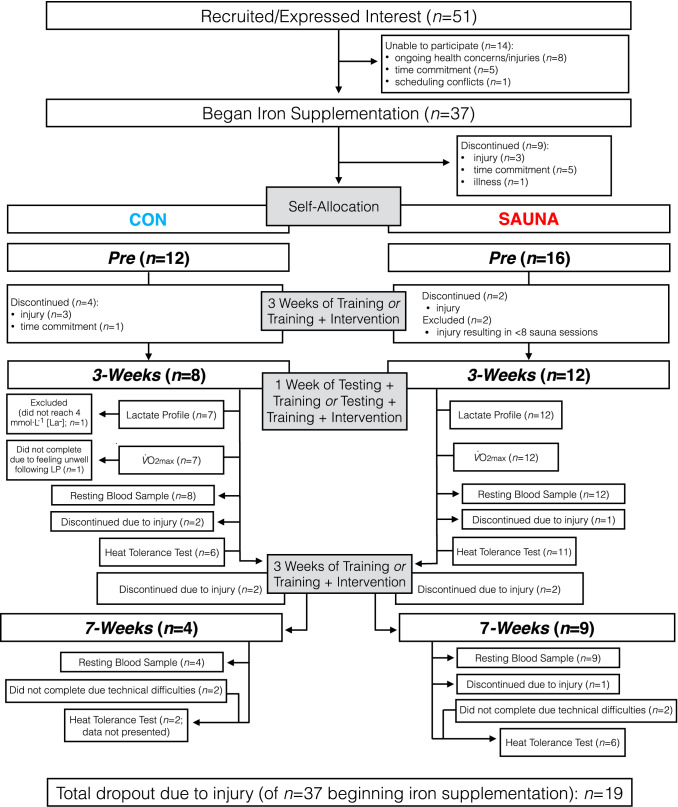
Progression of participants through phases of the study. *LP* lactate profile test, *[La*^*−*^*]* blood lactate concentration; $V$*O*_*2max*_ maximal aerobic capacity

Fifty-one trained middle-distance and cross-country runners were recruited from the university’s athletics club to participate in this study. However, due to various reasons (injury, scheduling, etc.) only 20 athletes (SAUNA, *n* = 12 [F, *n* = 9; M, *n* = 3]; CON, *n* = 8 [F, *n* = 4; M, *n* = 4]) completed experimental trials at 3-Weeks. Two female participants completed both the SAUNA and CON interventions (in opposite order, with ~ 5 weeks washout), one ‘cross-over’ participant only completing the Lactate Profile test at both Pre and 3-Weeks whilst undergoing the CON intervention. Eight athletes completed experimental trials at 7-weeks, though only the six athletes in the SAUNA group (F, *n* = 3; M, *n* = 3) will be reported in the HTT at the final time-point (7-Weeks). The two athletes in the CON group who completed experimental trials at 7-Weeks were not included in the 7-Weeks analysis. An overview of the progression of athletes through the study is detailed in Fig. 2. Participants in the SAUNA and CON group at 3-Weeks had similar baseline characteristics (age 19 ± 1 and 20 ± 2 years, height 169 ± 7 and 172 ± 6 cm, $\dot{V}$O_2max_ 59.3 and 58.4 ± 7.2 ml kg^−1^ min^−1^, respectively; all *p* > 0.05). SAUNA participants who completed experimental trials at 7-Weeks were 19 ± 1 years of age, 171 ± 6 cm tall and had a baseline $\dot{V}$O_2max_ of 60.2 ± 10.1 ml kg^−1^ min^−1^. Female participants included in analyses at 3-Weeks were eumenorrheic (*n* = 7), oligomenorrheic (*n* = 1), amenorrheic (*n* = 1), or using various forms of hormonal contraceptives (monophasic oral contraceptive pill, *n* = 1; progestin-only oral contraceptive pill, *n* = 1; contraceptive coil, *n* = 1). Of these, female participants included in analysis at 7-Weeks were either eumenorrheic (*n* = 2) or amenorrheic (*n* = 1). Female participants did not report any negative menstrual or Pre-menstrual symptoms that may have affected performance (Giacomoni et al. 2000).

### Post-exercise sauna bathing

Participants entered the sauna (101–108 °C at ~ 1.8 m, 5–10% RH at ~ 1 m; iButton Hygrochron Logger, Maxim Integrated, California, USA) within ~ 5 min of cessation of outdoor exercise. Sauna bathing typically followed low-intensity, continuous exercise training sessions (i.e., ‘easy runs’ or ‘long runs’). Participants were asked to remain in the sauna for 30-min, as per previous investigations (Scoon et al. 2007; Stanley et al. 2015). In the sauna, participants sat upright and were allowed to drink ad libitum. Peak heart rate (HR) was recorded during the final minute of exposure.

### Experimental trials

Participants performed all experimental trials at the same time of day (± 2 h). Participants were asked to recall the food they had eaten prior to experimental tests, and to repeat the same diet when re-tested. Participants voided their bladder upon arrival to the laboratory to provide a urine sample, which was analysed for osmolality. If urine osmolality was ≥ 700 mOsm kg^−1^ (Osmocheck, Vitech Scientific Ltd., West Sussex, United Kingdom; Sawka et al. 2007), participants drank 250 mL of water and did not begin exercising for at least 20 min.

### Running heat tolerance test (HTT)

The running heat tolerance test (HTT) was performed in hot conditions (40 °C, 40% RH) in an environmental chamber (TIS Services, Hampshire, United Kingdom) with a fan-generated airflow of ∼ 4 m s^−1^. Towel-dried, nude body mass was recorded to 0.1 kg using digital scales (Seca 877, Seca, Hamburg, Germany) before and immediately after the HTT to estimate whole-body sweat loss. Participants wore socks, shoes, and either shorts (males) or shorts and a sports bra (females). Participants ran on a treadmill (H/P/Cosmos Quasar 4.0, H/P/Cosmos, Germany) at 9 km h^−1^ and 2% gradient for 30 min (Mee et al. 2015). Rectal temperature (*T*_rec_), skin temperature (*T*_sk_), and HR were measured continuously. Perceptual measures, which included ratings of perceived exertion (RPE), thermal comfort and thermal sensation, were obtained in the final min of the HTT. Sweat gland activity of the forearm and upper back were recorded immediately following the HTT. Drinking was not permitted during the test.

### Lactate profile test

The lactate profile test was performed in temperate conditions (~ 18 °C). Participants completed a light 10-min warm-up before commencing the step-style incremental test on the treadmill. Participants completed 3-min stages with 30-s stops between stages when capillary [La^−^] was measured. The test began at 1% gradient (Jones and Doust 1996) and a speed 4 km h^−1^ slower than a recent 5-km race pace, and increased by 1 km h^−1^ for each stage thereafter. HR and respiratory gas exchange were continuously measured. The test was terminated when [La^−^] exceeded 4 mmol L^−1^, which occurred following 4–7 stages.

### Maximal aerobic capacity ($V$O_2max_)

Approximately 10 min after completing the lactate profile test, participants performed a ramp-style $V$O_2max_ test. The test began at 1% gradient and at the speed 2 km h^−1^ slower than the speed at which the participant had surpassed 4 mmol L^−1^ [La^−^]. Speed increased 1 km h^−1^ each minute for the next 2 min, when the speed at which > 4 mmol L^−1^ [La^−^] had been reached. At this point, the gradient was increased by 1% each minute until volitional exhaustion. Participants were given consistent and loud encouragement during the test. When participants were re-tested at 3-Weeks, the starting speed and progression of the protocol was repeated to allow for measurement of time-to-exhaustion (TTE). HR and respiratory gas exchange were continuously measured. Capillary [La^−^] was measured 5 min from the time of exhaustion.

### Resting blood sampling and analysis

On a separate visit (between the hours of 9:00–11:00 h), participants rested in a supine position for a minimum of 10 min, at which time resting HR was assessed and venous blood was drawn from the antecubital vein into a K2EDTA-coated vacutainer for measurement of plasma VEGF and an uncoated vacutainer for measurement of serum EPO. Vacutainers were kept on ice (plasma) or at room temperature (serum) for 45 min before centrifugation (4 °C, 10,000 rpm, 10 min) (Heraeus Multifuge X1R, Thermo Scientific, Loughborough, UK) and were frozen at − 80 °C until analysis. Plasma VEGF (Human VEGF DuoSet ELISA; R&D Systems, Abingdon, UK) and serum EPO (Human Erythropoietin DuoSet ELISA; R&D Systems, Abingdon, UK) were determined with ELISA kits.

### Measures

*T*_rec_ was measured using a rectal thermistor inserted 10 cm past the anal sphincter (Mon-a-Therm, Covidien, Mansfield, MA, United States). Weighted mean skin temperature was recorded using skin thermistors (Squirrel Thermal Couples, Grant Instruments, Cambridge, UK) attached to four sites on the left side of the body: pectoralis major (*T*_chest_), biceps brachii (*T*_arm_), rectus femoris (*T*_thigh_), and gastrocnemius lateral head (*T*_lowerleg_). Skin and rectal temperatures were continuously logged at 30-s intervals (Squirrel 2020 series, Eltek, Ltd., United Kingdom). HR (Polar Electro, Kempele, Finland) was recorded continuously (sampling rate of 1 Hz) on the Polar Beat application (Polar Beat, Kempele, Finland).

Active sweat glands were quantified using a modified-iodine paper technique with blinded computer aided analysis (Gagnon et al. 2012). Samples were collected by the same researcher from the dorsal side of the thickest segment of the forearm and mid-scapula on the upper back. Training session RPE was measured using a 1–10 point scale (RPE_1–10_; ‘very light session’ to ‘max effort session’) and RPE during HTTs was measured using the 6–20 point Borg Scale (RPE_6–20_; Borg 1982). Thermal sensation and thermal comfort were measured using modified 13-point and 10-point scales, respectively (Gagge et al. 1967). Measures of [La^−^] were taken from a fingertip capillary sample and immediately analysed using a Biosen C-Line Lactate analyser (EKF Diagnostics, Penarth, UK), which was quality checked each day and calibrated every 60 min. Respiratory gas exchange was sampled breath-by-breath using open-circuit spirometry (Vyntus CPX, Jaeger, Wuerzberg, Germany).

### Data analysis

Peak *T*_rec_ was the highest *T*_rec_ value measured during the HTT. *T*_recRISE_ was calculated as the change in *T*_rec_ from 0–30 min during the HTT. *T*_sk_ was calculated as a weighted average according to Ramanathan (1964):

$${T}_{\mathrm{sk}}=0.3\times \left({T}_{\mathrm{chest}}+{T}_{\mathrm{arm}}\right)+0.2\times ({T}_{\mathrm{thigh}}+{T}_{\mathrm{lower} \mathrm{leg}})$$

Estimated sweat loss was calculated as the difference between Pre- and post-HTT nude body mass. Running speed at 4 mmol L^−1^ [La^−^] was determined using a custom Matlab script (The Mathworks Inc, Natick, USA) to fit a third-order polynomial curve to each individual dataset and interpolate the running speed at 4 mmol L^−1^. Respiratory gas exchange data were exported as 5-s values and used to calculate oxygen consumption, running economy and respiratory exchange ratio (RER) during the lactate profile and $V$O_2max_ tests. Running economy (mL kg^−1^ km^−1^) was calculated as oxygen consumption (mL kg^−1^ min^−1^) divided by treadmill speed (km h^−1^) (Barnes and Kilding 2015). During the lactate profile test, submaximal RER, running economy and HR data were averaged across the final minute of the stage where participants exceeded 4 mmol L^−1^ [La^−^] at baseline, with the same running speed compared at 3-Weeks. $V$O_2max_ was calculated as the highest rolling 30-s average attained during the test. Successful attainment of $V$O_2max_ required meeting two of the following three criteria: (1) [La^−^] ≥ 8 mmol L^−1^ (Howley et al. 1995), (2) RER ≥ 1.10 (Edvardsen et al. 2014), (3) maximal HR ≥ 90% of age-predicted maximal HR (220 − age). Additionally, a plateau was confirmed both visually and systematically by a 5-s average value ≥ 2 standard deviations lower than the linear forecast of $V$O_2_ increase. For individual *T*_sk_ sites, if a time-point was lost, the missing data were modelled according to the slope/pattern of other data points from that participant during the previous test. Data at multiple *T*_sk_ sites were lost for two SAUNA group participants in the HTTs at 7-Weeks, and therefore SAUNA *T*_sk_ at 7-Weeks (*n* = 4) was deemed incomplete and not reported herein. Training data and resting HR were not collected in *n* = 1 SAUNA participant for both 3- and 7-Weeks.

### Statistical approach

All data were analysed using SPSS statistical software (SPSS version 25.0.0, SPSS, Chicago, IL, United States). To address the main aims, physiological data from HTT, lactate profile and $V$O_2max_ tests were compared between SAUNA and CON groups using a one-way analysis of co-variance (ANCOVA), with change scores (∆; difference between Pre and 3-Weeks) entered as the dependent variable and Pre-intervention absolute data entered as a co-variate. This allowed all changes to be covariate-adjusted for the baseline values (Vickers and Altman 2001) and yielded a single *p* value to represent the between-group comparison. This *p* value reflects the effect of the intervention. Because sex affects cardiorespiratory fitness, sex as a variable was tested for violations related to collinearity and entered as co-variate in analyses of $V$O_2max_ and running speed at 4 mmol L^−1^ [La^−^]. Effect sizes derived from between-group comparisons are presented as Cohen’s *d*, whereby *d* > 0.8 are considered large, 0.5–0.8 moderate and 0.2–0.5 small. Normality of these data was assessed using Levene’s test of equality of error variances, which was not violated on any occasion.

To address the subsidiary aim, a one-way repeated-measures analysis of variance (ANOVA) was used to compare HTT physiological data from the SAUNA group participants at Pre, 3-Weeks and 7-Weeks (time [3 levels]).

Total training frequency and average weekly running distance were compared either between groups (3-Weeks) using an independent *t* test (group [2 levels]) or within-group (7-Weeks) using a one-way repeated-measures ANOVA (week [7 levels]). Average weekly frequency of each type of training session (i.e., easy run, tempo training, interval training, and long run) were compared using a two-way ANOVA (3-Weeks: between groups, type [4 levels] × group [2 levels]; 7-Weeks: repeated-measures, type [4 levels] ×  week [7 levels]).

Normality of data analysed by ANOVA was assessed using Mauchly’s test of sphericity, and Greenhouse–Geisser corrections were applied where assumptions of sphericity were violated. When a significant main effect was found, Dunn-Bonferroni-corrected post hoc comparisons were made. Main effect sizes for ANOVAs were calculated using partial eta-squared (*η*_*p*_^2^), whereby *η*_*p*_^2^ > 0.14 are considered large, 0.06–0.14 moderate and < 0.06 small (Cohen 1988).

Ordinal data, including average running session RPE_1–10_, and peak RPE_6–20_, thermal comfort and thermal sensation during HTTs, were compared between groups (3-Weeks) using a Mann–Whitney *U* test or within-group (7-Weeks) using a Friedman’s test with post hoc analysis by Wilcoxon sign-rank tests.

Absolute data and within-subject changes are expressed as mean ± standard deviation (SD), and 95% confidence limits ([95% CL: lower limit, upper limit]) are presented with mean between-group differences. Significance was set at *p* < 0.05 for each analysis.

## Results

### Training

**Fig. 3:**
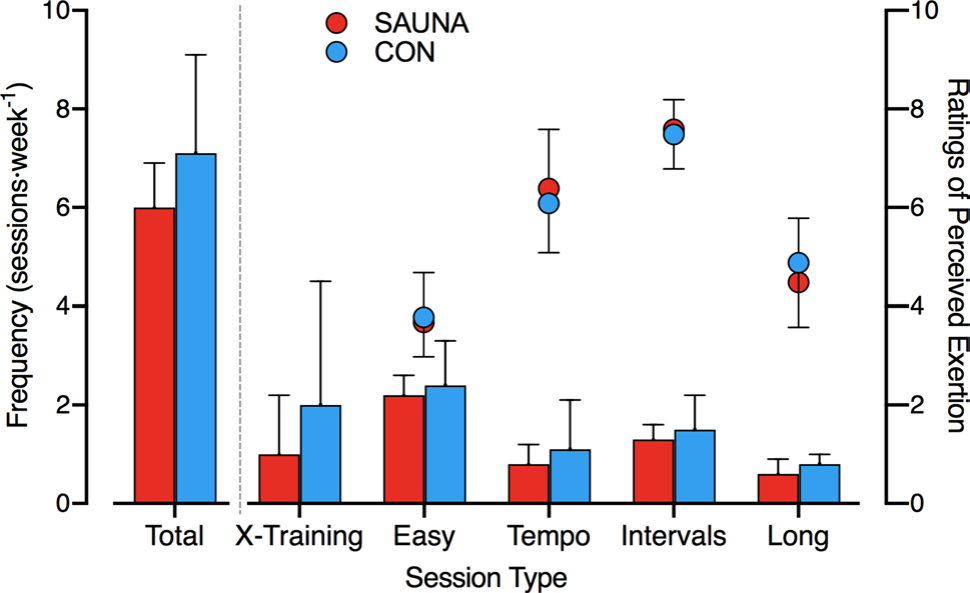
Training frequency, type (bars, left axis) and session perceived exertion (10-point scale; circles, right axis) for three-weeks training in the post-exercise sauna intervention (SAUNA, red) and control (CON, blue) groups. X-training, cross-training. Data are presented as mean ± SD

*3-Weeks:* Training sessions were performed 7 ± 2 times per week (SAUNA: 6 ± 1; CON: 7 ± 2) in the weeks between Pre and 3-Weeks testing. Average weekly running distances were 53.3 ± 23.3 km and 54.5 ± 28.2 km in SAUNA and CON, respectively. Training was not different in type, frequency, running distance or perceived exertion between the SAUNA and CON groups at 3-Weeks (all *p* > 0.05; Fig. 3).

*7-Weeks:* Participants in the SAUNA group trained 6 ± 1 times per week, and ran 59.1 ± 21.8 km week^−1^_._ Training was not different in type, frequency, running distance or perceived exertion across the 7 weeks prior to 7-Weeks testing in the SAUNA group (all *p* > 0.05).

### Post-exercise sauna bathing sessions

*3-Weeks:* Participants in the SAUNA group attended sauna sessions 3 ± 1 times per week, accumulating 9 ± 1 sauna sessions before completing the lactate profile and $V$O_2max_ tests, and 10 ± 1 sauna sessions before completing the HTT. Participants remained in the sauna for 28 ± 2 min each session, totalling 290 ± 48 min of sauna exposure. Peak HR reached 127 ± 10 beats min^−1^ whilst in the sauna.

*7-Weeks:* Participants in the SAUNA group attended sauna sessions 3 ± 1 times per week, accumulating 19 ± 1 sauna sessions before completing the final HTT at 7-Weeks. These participants remained in the sauna for 28 ± 1 min each session, totalling 528 ± 29 min of sauna exposure. Peak HR reached 120 ± 14 beats min^−1^ whilst in the sauna. Frequency, duration, and peak HR were not different between the sauna sessions completed before 3-Weeks testing as compared to those completed between 3-Weeks and 7-Weeks testing (all *p* > 0.05).

### Menstrual cycle

Female participants completed all experimental trials at Pre, 3-Weeks and 7-Weeks 28 ± 2 days apart. Normally menstruating participants (*n* = 8) reported menstrual cycles ranging between 26–31 days and five completed all tests in the same phase of their menstrual cycle. Of the remaining three, one SAUNA participant completed all Pre tests in the follicular phase and all 3-Weeks tests in the luteal phase, one SAUNA participant completed temperate exercise tests at Pre in the luteal phase and at 3-Weeks in the follicular phase (though HTTs were completed in the same phase), one CON participant completed temperate exercise tests at Pre in the follicular phase and at 3-Weeks in the luteal phase (HTTs completed in the same phase). Participants using oral contraceptives (*n* = 2) completed all tests in the active pill-taking phase.

### Resting measures

*3-Weeks*: Changes in resting HR were not different between groups at 3-Weeks as compared to Pre (SAUNA: 58 ± 6 vs 55 ± 9 beats min^−1^; CON: 54 ± 6 vs 51 ± 4 beats min^−1^, at Pre vs 3-Weeks, respectively; *p* = 0.90, *d* = 0.06). Changes in body mass were not different between groups (SAUNA: 61.0 ± 6.3 vs 61.2 ± 6.4 kg; CON: 63.8 ± 5.5 vs 63.5 ± 5.4 kg, at Pre vp 3-Weeks, respectively; *p* = 0.43, *d* = 0.45). Serum EPO was detectable in 17 out of 20 samples (SAUNA, *n* = 12; CON, *n* = 5). Changes in resting serum EPO were not different (*p* = 0.82, *d* = 0.24) between groups (SAUNA: 3.8 ± 2.7 vs 3.1 ± 1.6 mIU mL^−1^; CON: 2.8 ± 2.3 vs 2.6 ± 1.9 mIU mL^−1^, at Pre vs 3-Weeks, respectively). VEGF was detectable in 10 out of 20 samples (SAUNA, *n* = 5; CON, *n* = 5). Changes in resting plasma VEGF were not different (*p* = 0.09, *d* = 0.35) between groups (SAUNA: 128.5 ± 121.9 vs 117.9 ± 106.4 pg mL^−1^; CON: 183.0 ± 234.4 *vs* 179.8 ± 274.7 pg mL^−1^, at Pre vs 3-Weeks, respectively).

*7-Weeks*: Participants in the SAUNA group showed a trend (*p* = 0.06) for a decrease in resting HR across time, though this was not significant (57 ± 6, 53 ± 10 and 53 ± 7 beats min^−1^ at Pre, 3-Weeks and 7-Weeks, respectively). Serum EPO did not significantly change across time (3.6 ± 1.6, 3.1 ± 1.8 and 4.4 ± 2.8 mIU mL^−1^ at Pre, 3-Weeks and 7-Weeks, respectively; *p* = 0.18). VEGF was detectable in *n* = 3 samples and therefore not statistically compared (77.9 ± 55.6, 77.5 ± 39.4 and 76.1 ± 51.6 pg mL^−1^ at Pre, 3-Weeks and 7-Weeks, respectively).

### Running heat tolerance test

#### Body temperatures

**Fig. 4:**
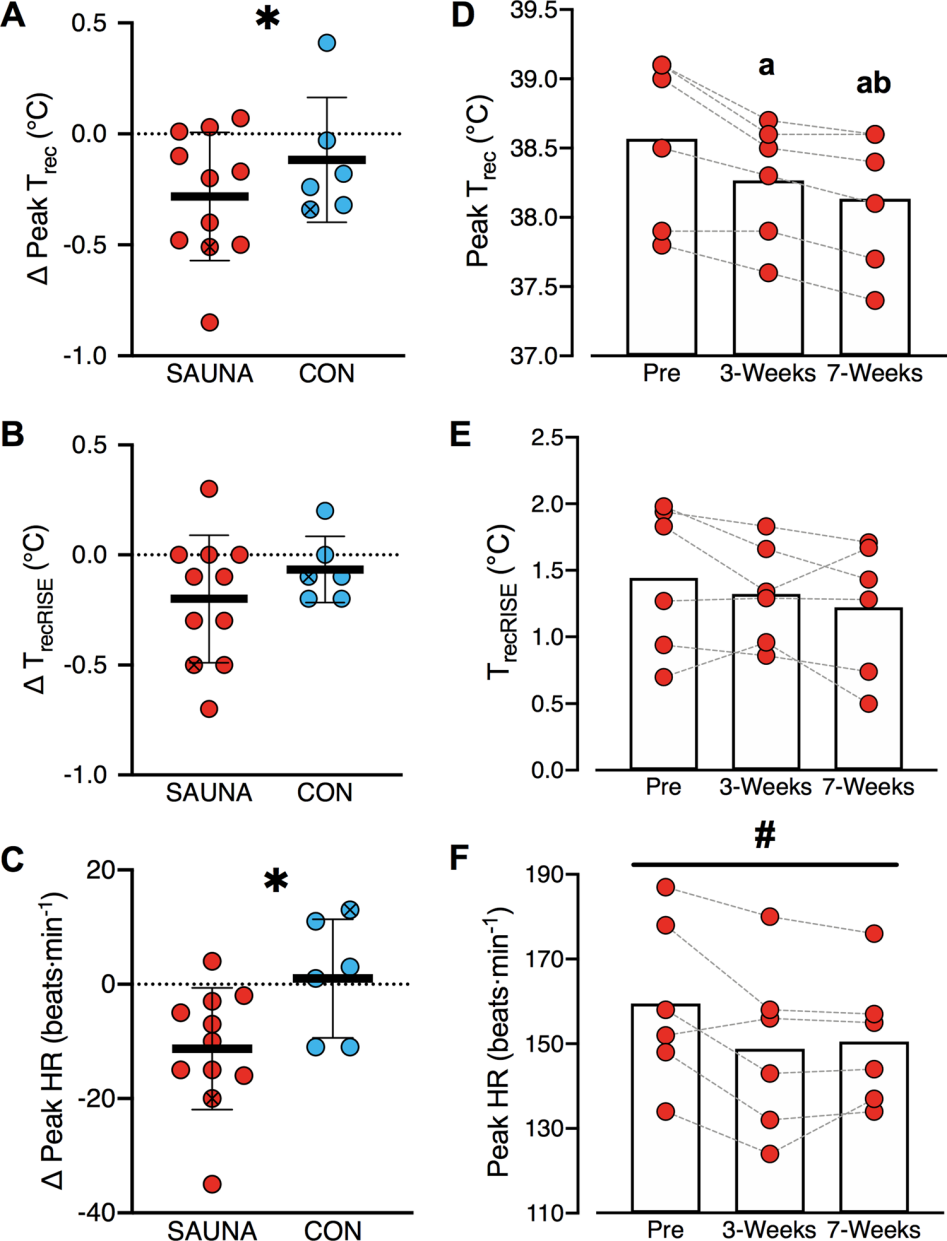
Delta (Δ) peak rectal temperature (*T*_rec_; **a**), rise in rectal temperature (*T*_recRISE_; **b**) and peak heart rate (HR; **c**) during the running heat tolerance test (HTT; 40 °C, 40%RH) in the SAUNA (red) and CON (blue) groups from baseline (Pre) to following three-weeks post-exercise sauna bathing intervention or control (3-Weeks). Absolute peak Trec (**d**), TrecRISE (**e**) and peak heart rate (**f**) during the HTT at Pre, 3-Weeks and following seven-weeks intervention (7-Weeks). Horizontal black lines (**a**–**c**) or bars (**d**–**f**) and error bars represent the group mean ± SD. Circles represent individual participant data. The participant who completed both SAUNA and CON interventions is indicated by an *X*. *Significant difference in SAUNA vs CON; ^#^significant main effect of time; ^a^significant difference from Pre; ^b^significant difference from 3-Weeks, (*p* < 0.05)

*3-Weeks:* Body temperature responses at Pre and 3-Weeks are detailed in Table 1. The SAUNA group exhibited a 0.2 °C [0.0, 0.4] greater reduction in peak *T*_rec_ than the CON group at 3-Weeks as compared to Pre (*p* = 0.04, *d* = 0.67; Fig. 4a). However, changes in *T*_recRISE_ were not different between groups (*p* = 0.14; *d* = 0.54; Fig. 4b). The SAUNA group exhibited a 0.8 °C [0.1, 1.5] greater reduction in peak *T*_sk_ than the CON group at 3-Weeks as compared to Pre (*p* = 0.03, *d* = 0.99).

**Table 1:** Physiological and perceptual responses during the running Heat Tolerance Test

	SAUNA		CON	
	Pre	3-Weeks	Pre	3-Weeks
Peak *T*_rec_ (°C)	38.6 ± 0.5	38.3 ± 0.3*	38.6 ± 0.4	38.5 ± 0.3
*T*_recRISE_ (°C)	1.4 ± 0.4	1.2 ± 0.3	1.5 ± 0.5	1.5 ± 0.5
Peak *T*_sk_ (°C)	36.4 ± 1.3	35.7 ± 0.8*	36.3 ± 1.4	36.5 ± 1.2
Peak HR (beats min^−1^)	163 ± 24	152 ± 19*	157 ± 16	158 ± 21
Sweat loss (kg)	0.8 ± 0.2	0.8 ± 0.2*	0.8 ± 0.3	0.7 ± 0.1
Forearm active sweat glands (per cm^2^)	51 ± 24	70 ± 20*	45 ± 20	44 ± 24
Upper back active sweat glands (per cm^2^)	64 ± 15	70 ± 24	71 ± 16	63 ± 26
Peak RPE_6–20_	12 ± 2	10 ± 2	11 ± 2	11 ± 2
Peak thermal sensation	10 ± 1	9 ± 1*	9 ± 1	9 ± 0
Peak thermal comfort	5 ± 2	4 ± 2*	3 ± 2	4 ± 2

Data are presented as mean ± SD

*HR* heart rate, *T*_*rec*_ rectal temperature, *T*_*recRISE*_ change in rectal temperature during exercise, *RPE*_*6–20*_ rating of perceived exertion (6–20 point scale). Thermal sensation and thermal comfort were measured on 13- and 10-point scales, respectively

*Change from Pre to 3-Weeks in SAUNA group significantly different from CON (*p* < 0.05)

*7-Weeks:* Peak *T*_rec_ in the SAUNA group showed a large and significant main effect of *time* (*p* < 0.01, *η*_*p*_^2^ = 0.84; Fig. 4d). Post hoc analysis revealed that peak *T*_rec_ was 0.3 °C [0.0, 0.6] lower in SAUNA at 3-Weeks (*p* = 0.04) and 0.4 °C [0.2, 0.6] lower at 7-Weeks (*p* < 0.01), as compared to Pre. Furthermore, peak *T*_rec_ was 0.1 [0.0, 0.3] °C lower at 7-Weeks as compared to 3-Weeks (*p* = 0.03).

#### Heart rate

*3-Weeks:* The SAUNA group exhibited an 11 beats min^−1^ [0, 22] greater reduction in peak HR during the HTT than the CON group at 3-Weeks as compared to Pre (*p* < 0.05, *d* = 1.16; Fig. 4c; Table 1).

*7-Weeks:* Peak HR in the SAUNA group showed a large and significant main effect of *time* (*p* = 0.02, *η*_*p*_^2^ = 0.54; Fig. 4f). Post hoc analysis showed no significant differences, however there was a trend for peak HR to be lower at 3-Weeks as compared to Pre (− 11 beats min^−1^ [+ 2, − 23]; *p* = 0.08).

#### Sweating

*3-Weeks:* Absolute sweating data at Pre and 3-Weeks are detailed in Table 1. The CON group showed an attenuated sweat loss of 0.2 kg [0.0, 0.3] during the HTT, as compared to the SAUNA group at 3-Weeks vs Pre (*p* = 0.02, *d* = 0.95). The SAUNA group exhibited a 54 ± 59% increase in sweat gland activity on the forearm, equating to a 22 active glands per cm^2^ (Cohen 1988; Racinais et al. 2015b) greater increase than the CON group’s 6 ± 17% reduction, at 3-Weeks as compared to Pre (*p* < 0.01, *d* = 1.40). Changes in sweat gland activity on the upper back were not different between groups (*p* = 0.59, *d* = 0.35).

*7-Weeks:* Sweat loss in the SAUNA group was not different between any of the HTTs (0.9 ± 0.2, 0.8 ± 0.1 and 0.8 ± 0.2 kg at at Pre, 3-Weeks and 7-Weeks, respectively; *p* = 0.21, *η*_*p*_^2^ = 0.27). Sweat gland activity on the forearm showed a large and significant main effect of time (48 ± 17, 66 ± 14 and 69 ± 13 active glands per cm^2^ at Pre, 3-Weeks and 7-Weeks, respectively; *p* = 0.01, *η*_*p*_^2^ = 0.57). Post hoc analysis showed no significant differences, however there was a trend for sweat gland activity on the forearm to be greater at 3-Weeks as compared to Pre (*p* = 0.07). Sweat gland activity on the upper back was not different between any of the HTTs (72 ± 6, 77 ± 20 and 75 ± 7 active glands per cm^2^ at Pre, 3-Weeks and 7-Weeks, respectively; *p* = 0.33, *η*_*p*_^2^ = 0.20).

#### Perceptual measures

*3-Weeks:* Perceptual data at Pre and 3-Weeks are detailed in Table 1. The SAUNA group exhibited a 1 ± 1 scale point greater reduction in peak thermal sensation (*p* = 0.02) and a 2 ± 2 scale point greater reduction in peak thermal comfort (*p* < 0.01) during the HTT than the CON group at 3-Weeks as compared to Pre. There was also a trend (*p* = 0.08) for a 2 ± 2 scale point greater reduction in RPE_6–20_ during the HTT in the SAUNA group than the CON group at 3-Weeks as compared to Pre.

*7-Weeks:* Peak RPE in the SAUNA group was significantly different between the HTTs (main effect: *p* = 0.04; 12 ± 3, 11 ± 2 and 10 ± 2 at Pre, 3-Weeks and 7-Weeks, respectively). Post hoc analysis revealed peak RPE was lower at 7-Weeks as compared to Pre (*p* = 0.04) and as compared to 3-Weeks (*p* = 0.03). There was a trend (*p* = 0.08) for a reduction in peak RPE at 3-Weeks as compared to Pre. Thermal sensation was not different between HTTs (main effect: *p* = 0.12; 10 ± 1, 9 ± 1 and 9 ± 1 at Pre, 3-Weeks and 7-Weeks, respectively). Similarly, thermal comfort was not different between HTTs (main effect: *p* = 0.09; 5 ± 2, 4 ± 2 and 3 ± 1 at Pre, 3-Weeks and 7-Weeks, respectively).

### Lactate profile test

**Fig. 5:**
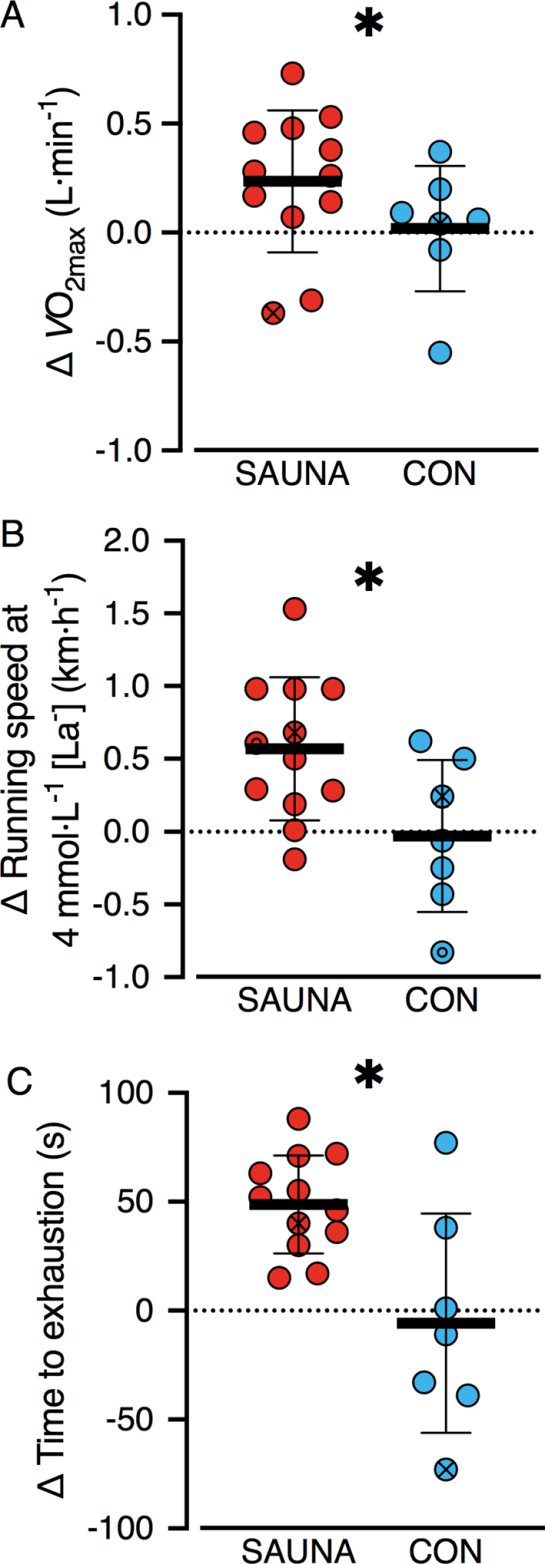
"Delta (Δ) exercise performance markers in temperate conditions (18 °C) at baseline (Pre) to following three-weeks (3-Weeks) post-exercise sauna bathing intervention (SAUNA; red, left panel) or control (CON; blue, right panel). Mean ± SD (horizontal lines and error bars) plus individual participant data (circles) presented for delta (Δ) maximal aerobic capacity ($V$O_2max_, **a**), running speed at 4 mmol L^−1^ blood lactate concentration ([La^−^]) (**b**) and running time to exhaustion (**c**). The two participants who completed experimental tests following both SAUNA and CON interventions as indicated by an *X* and (in **b** only) an *O*. *Significant difference in SAUNA vs CON (*p* < 0.05)

Physiological responses during the lactate profile test at Pre and 3-Weeks are detailed in Table 2. The SAUNA group exhibited a 4 ± 3% improvement in running speed at 4 mmol L^−1^ [La^−^], equating to a 0.6 km h^−1^ [0.1, 1.0] greater improvement than the CON group (0 ± 3%) at 3-Weeks as compared to Pre (*p* = 0.01, *d* = 1.19; Fig. 5b).

**Table 2:** Physiological responses to temperate exercise tests

	SAUNA		CON	
	Pre	3-Weeks	Pre	3-Weeks
$V$O_2max_ (L min^−1^)	3.42 ± 0.81	3.65 ± 0.75*	3.72 ± 0.76	3.73 ± 0.55
TTE (s)	426 ± 57	474 ± 59*	439 ± 62	433 ± 79
Running speed at 4 mmol L^−1^ [La^−^] (km h^−1^)	15.6 ± 1.7	16.2 ± 1.7*	16.8 ± 1.5	16.8 ± 1.4
Submaximal HR (beats min^−1^)	188 ± 9	183 ± 8	178 ± 12	182 ± 12
Submaximal RER	1.01 ± 0.06	1.00 ± 0.05	1.02 ± 0.03	1.04 ± 0.03
Submaximal RE (mL kg^−1^ min^−1^)	189 ± 14	195 ± 11	187 ± 25	194 ± 22

Data are presented as mean ± SD

$V$*O*_*2max*_ maximal aerobic capacity, *TTE* time to exhaustion, *[La*^*−*^*]* blood lactate concentration, *HR* heart rate, *RER* respiratory exchange ratio, *RE* running economy

*Change from Pre to 3-Weeks in SAUNA group significantly different from CON (*p* < 0.05)

During the lactate profile test, there was a trend (*p* = 0.06; *d* = 1.40) for a greater reduction in submaximal HR in the SAUNA group than in the CON group at 3-Weeks as compared to Pre (− 7 beats min^−1^ [0, − 14]). Neither changes in submaximal running economy (*p* = 0.73, *d* = 0.27) nor RER (*p* = 0.14, *d* = 0.30) were different between groups.

### Maximal aerobic capacity (${\varvec{V}}$O_2max_) test

The SAUNA group exhibited an 8 ± 12% improvement in $V$O_2max_, equating to a 0.27 L min^−1^ [0.05, 0.49] greater improvement than the CON group (+ 2 ± 8%) at 3-Weeks as compared to Pre (*p* = 0.02, *d* = 0.69; Fig. 5a; Table 2). Similarly, the SAUNA group exhibited a 12 ± 6% improvement in TTE during the $V$O_2max_ test, equating to a 54 s [17, 90] greater improvement than the CON group (− 1 ± 11%) at 3-Weeks as compared to Pre (*p* < 0.01, *d* = 1.56; Fig. 5c; Table 2).

## Discussion

The main findings of this study are that 3 weeks of post-exercise sauna bathing induced heat acclimation adaptations (reduced HR, *T*_rec_ and *T*_sk_, and increased sweat gland activity), and improved markers of exercise performance in temperate conditions (i.e., $V$O_2max_, running speed at 4 mmol L^−1^ [La^−^] and TTE), to a greater extent than endurance training alone. These improvements were consistent with the study hypothesis. Following 7 weeks of post-exercise sauna bathing, *T*_rec_ was the only physiological variable measured during the HTT that showed further improvements. However, the additional 0.1 °C reduction in peak *T*_rec_ from 3-Weeks to 7-Weeks may not be physiologically meaningful. Whilst participants did adhere well to the intervention, the dropout rate was high (51%; mainly due to injuries acquired during normal training in both groups). This drop-out rate was an important finding in-and-of-itself as it highlights the difficulty of implementing a 7-week long heat acclimation protocol in addition to the training programme of competitive endurance runners.

### Exercise heat tolerance

The mean 0.3 °C reduction in peak *T*_rec_ observed in the SAUNA group at 3-Weeks surpasses the coefficient of variation of the HTT (0.34%, corresponding to 0.1 °C in the current dataset) as validated by Mee and colleagues (2015). Furthermore, the 0.2 °C difference in the change in peak *T*_rec_ between the SAUNA and the CON groups meets the physiologically meaningful threshold identified in a meta-analysis by Tyler and colleagues (2016). This reduction in peak *T*_rec_ in the SAUNA group may have resulted from an increased heat dissipation, possibly by means of increased skin blood flow combined with greater peripheral sweat gland activation to improve evaporative heat loss efficiency (Taylor 2014; Smith and Havenith 2019), given we did not observe increased whole-body sweat loss. The 0.8 °C greater reduction in peak *T*_sk_ observed in the SAUNA group at 3-Weeks as compared to the CON group is consistent with other heat acclimation interventions, as Tyler et al. (2016) reported a 0.5 ± 0.5 °C reduction in peak *T*_sk_ across 67 interventions. Similarly, the 11 beats min^−1^ greater reduction in peak HR observed in the SAUNA group at 3-Weeks as compared to the CON group is consistent with other heat acclimation interventions, which report a 16 ± 6 beats min^−1^ reduction in peak HR across 118 interventions (Tyler et al. 2016). This reduction in peak HR in the SAUNA group was likely mediated by increased plasma and/or blood volume, as was demonstrated using a similar post-exercise sauna bathing protocol by Scoon et al. (2007), though these variables were not measured in the current study. Though endurance training may (Nadel et al. 1974; Ichinose et al. 2009; untrained cohorts, 10 days to ~ 3 months training) or may not (McGarr et al. 2014; moderately-trained cohort, 2 week training) improve thermoregulation during exercise heat stress, the changes in *T*_rec_, *T*_sk_ and HR in the SAUNA group were greater than those observed in the CON group. Overall, this indicates that the improvements observed in the SAUNA group during the HTT were as a result of the post-exercise sauna intervention as opposed to normal training. Therefore, intermittent post-exercise sauna bathing appears to induce thermoregulatory and cardiovascular adaptations that are superior to 3-weeks normal training, and of a similar magnitude to those observed in other heat acclimation protocols in the literature (Tyler et al. 2016). These improvements occurred despite using only ten 30-min exposures and did not cause any disruptions to normal training.

Sweat loss during the HTT differed between groups. In the CON group, there was a reduction in sweat loss during the HTT at 3-Weeks. Conversely, there was no change in sweat loss in the SAUNA group during the HTT at 3-Weeks, despite their lower body temperature (i.e., *T*_re_ and *T*_skin_). This indicates a possible seasonal change in sweat rate for the CON group (Matsumoto et al. 1990), whereas the SAUNA group showed an enhanced evaporative heat loss for a given body temperature. Further, at 3-Weeks, the SAUNA group exhibited a substantially greater increase (+ 54%) in sweat gland activation on the forearm than the CON group (− 6%). Though sweat gland activation is not indicative of sweat gland output, these findings are supported by a recent study that showed a greater relative sweat output on the limbs following heat acclimation (Smith and Havenith 2019). Notably, the increase in sweat gland activation on the forearm without increases in total sweat loss (as exhibited by the SAUNA group in the current study) may be a more efficient adaptation for athletes competing in hot-humid environments, as it would improve evaporative capacity without exacerbating dehydration (Alber-Wallerström and Holmér 1985).

Besides the further ~ 0.1 °C reduction in peak *T*_rec_ at 7-Weeks in the SAUNA group, physiological adaptations appeared to plateau by 3-Weeks, despite nearly doubling the participants’ total sauna exposures by 7-Weeks. This plateau is consistent with data from active heat acclimation interventions, whereby changes in heart rate and body temperatures are nearly maximised by ~ 7 days (Griefahn 1997; Periard et al. 2015). Interestingly, RPE_6–20_ was only decreased at 7-Weeks. Whether this would coincide with the anticipated steady increase in self-paced exercise capacity in the heat with a long-term intervention (Periard et al. 2015; Racinais et al. 2015b) is unknown, as the HTT was completed at a fixed workload.

### Temperate exercise performance

Our data demonstrate that 3 weeks of intermittent post-exercise sauna bathing is ergogenic for exercise performance in temperate conditions in well-trained participants. The training status of the athletes in the current study make these results especially meaningful. The 8% mean improvement in $V$O_2max_ observed in the current study (~ 6% more than the CON group) is comparable to the 5% observed by Lorenzo and colleagues (2010) when using a 10-day active heat acclimation protocol. Possible mechanisms facilitating this improvement in aerobic capacity with heat acclimation include a greater maximum cardiac output, as demonstrated by Lorenzo et al. (2010) in their study, or increases in total haemoglobin mass, which are possible in an intervention stretching over multiple weeks, as more recently demonstrated by Rønnestad et al. (2020). The iron supplements given to participants in the current study may have helped to facilitate improvements in oxygen carrying capacity, though participants were not given iron supplements by Rønnestad et al. (2020) or by Scoon et al. (2007), who both observed haematological adaptations following heat acclimation.

Three weeks of post-exercise sauna bathing also increased running speed at 4 mmol L^−1^ [La^−^] by ~ 4%, equating to a 0.6 km h^−1^ greater improvement than in the CON group. These results were similar to the 5% improvement in cycling power output at 4 mmol L^−1^ [La^−^] reported by Lorenzo et al. (2010), and are supported by other observations of reduced lactate accumulation during exercise following heat acclimation (Young et al. 1985). Although this improvement in running speed at 4 mmol L^−1^ [La^−^] was accompanied by a trend for a greater reduction in submaximal HR in the SAUNA group, running economy was not different during the lactate profile test at 3-Weeks. Thus, changes in [La^−^] may have been due to short-term adaptations such as a decreased rate of glycogenolysis (Febbraio et al. 1994; Kirwan et al. 1987), or due to better splanchnic perfusion (and thus improved lactate removal) and haemodilution via increased blood and plasma volume (Scoon et al. 2007; Lorenzo et al. 2010). Alternatively, the multi-week design of the current intervention might have allowed time for heat acclimation-induced mitochondrial biogenesis to manifest (Tamura et al. 2014; Hafen et al. 2018).

The SAUNA group’s ~ 12% increase in TTE following 3-Weeks post-exercise sauna bathing was superior to the CON group’s ~ 1% reduction in TTE. The mean improvement in the SAUNA group was lower than the 32% increase observed by Scoon et al. (2007), however there were important differences in the TTE tests used, making comparisons difficult. Scoon and colleagues implemented a ~ 15-min TTE test at the runners’ best 5-km race pace and 0% gradient. In the current study, the TTE test increased in either speed or gradient each minute and on average participants reached volitional exhaustion at ~ 8 min, but at a running speed at which they had reached 4 mmol L^−1^ [La^−^] and at a 4% incline. Despite using a different style of TTE test, this study builds on evidence for the ergogenic benefits of post-exercise sauna bathing provided by Scoon and colleagues, with the additions of the gold-standard test of aerobic capacity, a robust submaximal exercise test and a larger, mixed-sex cohort.

### Training and intervention

Of the 37 participants who began the experiment, 19 athletes succumbed to an injury (ranging from minor injuries that prevented training for ~ 1 week, to more severe injuries such as stress fractures or sprains) and did not complete the study. This is consistent with data reported in a review by van Gent et al. (2007), whereby incidences of lower extremity running injuries ranged between 19 and 79% in distance runners, with the highest rates observed in athletes reporting weekly running distances and training frequencies similar to those in the current study (i.e., females running 48–63 km week^−1^, males running 64+  km week^−1^, and athletes training 6–7 days week^−1^). For those who completed the intervention, training was consistent across the 7 weeks of post-exercise sauna bathing. Importantly, unlike active heat acclimation protocols that require changes to an athlete’s training to accommodate heat acclimation sessions, there were no differences in training between the CON group and SAUNA group in this study. This included similar perceived training intensity and training volume (i.e., frequency and type of sessions and weekly running distance) across the intervention period.

### Blood markers

Serum EPO changes in the SAUNA group were not significantly different from the CON group at 3-Weeks, and did not significantly change in the SAUNA group by 7-Weeks. As EPO is inherently variable in response to environmental stimuli (Chapman 2013; Płoszczyca et al. 2018), additional data may be required to fully understand this effect. Furthermore, serum EPO reflects a balance between renal EPO production and EPO consumption in the bone marrow (Rusko et al. 2004), making it difficult to discern the influence of either production or consumption by measurement of circulating EPO levels alone. Although the subsequent haematological responses of either increased consumption or production of EPO (i.e., increased red blood cell production) would coincide with the improved aerobic capacity observed at 3-Weeks in the current study and the increased red cell volume observed by Scoon et al. (2007), we were unable to measure red blood cell mass in the current study. VEGF was largely undetectable across the study, as a number of participant samples fell outside the ELISA kit’s limits of detection for the measurement, providing little insight into any possible angiogenesis with post-exercise sauna bathing.

### Perspectives and considerations

These results contribute to the growing body of research supporting favourable health and performance outcomes following passive heating interventions. The investigation of the efficacy of post-exercise sauna bathing to induce heat acclimation is especially timely as the Tokyo 2020 Olympic games (though postponed) are predicted to be the hottest Summer Olympic Games to date. We observed that heat acclimation was nearly maximised by three weeks of intermittent post-exercise sauna bathing and 7 weeks did not induce any further meaningful physiological improvements. Thus, athletes need only incorporate a post-exercise sauna regime for 3 weeks to achieve beneficial adaptations.

This study did not exclude participants based on menstrual cycle or hormonal contraceptive use, and instead included a random sample of female athletes in random phases of their menstrual cycle. The diverse menstrual cycle patterns and contraceptive choices observed in this cohort is representative of the female athlete population. Whilst menstrual cycle was self-reported, the study design successfully allowed for tests and re-tests to be in the same phase for most female participants, making it unlikely that results were skewed or biased by menstrual cycle.

Participants self-selected into the SAUNA or CON groups. However, participants in both groups were given performance feedback following temperate exercise tests, which they discussed with their coach and used to inform their training. Given the highly competitive nature of the athletes recruited and the training ethos of the running club they were recruited from, the SAUNA and CON groups were equally invested in their training and performance during the exercise tests. This is supported by the similar SAUNA and CON training profiles (intensity, frequency, type and duration). Furthermore, the objective nature of the $V$O_2max_ and lactate profile tests reduces the impact of extrinsic, motivational factors on temperate performance results.

Although alternative methods of lactate profile testing may be preferred for determining lactate threshold (i.e., maximal lactate steady-state, or the D-max method described by Cheng et al. 1992), these methods significantly underestimate ‘race pace’ for ~ 5-km races (Palmer et al. 1999; Chalmers et al. 2015). The training aims of the running club that participants were recruited from for the current study were largely centred around cross-country races (typically ranging between 4 and 6 km). Thus, running speed at 4 mmol L^−1^ [La^−^], observed to closely reflect 5-km race pace (Fay et al. 1989), had important ecological relevance for this cohort. In addition, though the results of this study certainly imply an improved exercise capacity, the extent at which the observed adaptations would impact self-paced performance in either the heat or in temperate conditions is unknown.

Finally, the repeated-measures aspect of the two participants who undertook both the SAUNA and CON intervention are not statistically accounted for. However, as depicted in figures where individual data from the cross-over participant(s) are indicated, this exerted a negative bias for most measures. A sensitivity analysis was undertaken whereby the cross-over data for the two cross-over participants was removed (e.g., participants’ data from the intervention they completed first were only included, and data from their cross-over intervention were removed; see supplementary material).

## Conclusion

To the best of our knowledge, this study is the first to demonstrate that post-exercise sauna bathing can induce thermoregulatory adaptations during exercise at an absolute fixed-workload, which supports current recommendations to use post-exercise sauna bathing as an alternative to active heat acclimation (Racinais et al. 2019). These adaptations occur following ~  10 intermittent exposures across three weeks. Extending the intervention to seven weeks appeared to have little additional benefit. Further, this study supports the efficacy of heat acclimation as an ergogenic aid for exercise performance in temperate conditions. Specifically, intermittent post-exercise sauna bathing on average improved $V$O_2max_ by ~ 8%, running speed at 4 mmol L^−1^ [La^−^] by ~ 4%, and time to exhaustion by ~ 12%, all of which were significantly greater improvements than those exhibited by the CON group. This intermittent-style intervention is especially attractive as the flexible nature and minimal impact on normal training circumvent common challenges associated with more traditional active heat acclimation approaches.

## Electronic supplementary material

Below is the link to the electronic supplementary material.

Supplementary file1 (DOCX 15 KB)

Supplementary file2 (XLSX 33 KB)

## Data Availability

All data generated or analysed during this study on which the conclusions of the paper rely are included in this published article (and its supplementary information files).

## Abbreviations

3-Weeks: Tests following three-weeks intervention
7-Weeks: Tests following seven-Weeks intervention
ANOVA: Analysis of variance
ANCOVA: Analysis of co-variance
CON: Control group
EPO: Erythropoietin
HR: Heart rate
HTT: Running heat tolerance test
Pre: Pre-intervention tests
RER: Respiratory exchange ratio
RPE: Rating of perceived exertion
SAUNA: Sauna intervention group
*T*_rec_: Rectal temperature
*T*_sk_: Mean weighted skin temperature
TTE: Time-to-exhaustion
VEGF: Vascular endothelial growth factor
*V*O_2max_: Maximal aerobic capacity
