# Supplementary material for: Intermittent post-exercise sauna bathing improves markers of exercise capacity in hot and temperate conditions in trained middle-distance runners
Source: Eur J Appl Physiol. 2020 Nov 19;121(2):621–35. doi: 10.1007/s00421-020-04541-z (PMC7862510; doi:10.1007/s00421-020-04541-z)
Supplement: Supplementary file 1 — Supplementary file1 (DOCX 15 KB) [file 421_2020_4541_MOESM1_ESM.docx]

**Intermittent post-exercise sauna bathing improves markers of exercise capacity in hot and temperate conditions in trained middle-distance runners**

Authors:

*Nathalie V. Kirby^1^

Samuel J. E. Lucas^1^

Oliver J. Armstrong^2^

Samuel R. Weaver^1^

Rebekah A. I. Lucas^1^

^1^University of Birmingham, Birmingham, United Kingdom

^2^Performance Centre, University of Birmingham Sport, Birmingham, United Kingdom

**Sensitivity Analysis**

A sensitivity analysis was performed on main outcome measures by removing the *n*=2 “cross-over” participants’ (i.e., participants who completed both SAUNA and CON interventions, with a ~5-weeks washout in-between) second intervention data set from the ANCOVA analyses (i.e., participant’s data from the intervention they completed first was *only* included).

After removing *n*=2 participants, the SAUNA group showed trends for greater reductions in peak HR (*p*=0.07, *d*=1.07) and peak T_rec_ (*p*=0.06, *d*=0.59) than the CON group. The CON group still showed an attenuated sweat loss (*p*=0.03, *d*=0.85) as compared to the SAUNA group. Consistent with our original analysis, the SAUNA group exhibited a greater improvement in running speed at 4 mmol·L^-1^ [La^-^] (*p*=0.05, *d*=0.94), $V$O_2max_ (*p*=0.02, *d*=0.96) and TTE (*p*<0.01, *d*=1.54) than the CON group.
